# Rush Medical College

**Published:** 1847-04

**Authors:** 


ARTICLE IV.

### RUSH MEDICAL COLLEGE.

At the recent commencement of this institution, the degree of Doctor of Medicine was conferred upon sixteen young gentlemen, who had undergone the necessary examination by the faculty, and complied with the requirements of the college.

The honorary degree was also conferred upon Dr. Samuel Grimes, of Delphi, Indiana.

We understand that a catalogue of students of the last, and the annual announcement for the ensuing session will soon appear.

